# AWG-Based Spectral Multiplexing for Unambiguous Range-Extended FMCW LiDAR

**DOI:** 10.3390/s26051435

**Published:** 2026-02-25

**Authors:** Sangwon Park, Sang Min Park, Seongmun Jeong, Gyeongmin Kweon, Chang-Seok Kim, Hwidon Lee

**Affiliations:** 1 Department of Optics and Mechatronics Engineering, Pusan National University, Busan 46241, Republic of Korea; sangaone@pusan.ac.kr (S.P.); smjeong99@pusan.ac.kr (S.J.); 2 Engineering Research Center for Color-Modulated Extra-Sensory Perception Technology, Pusan National University, Busan 46241, Republic of Korea; psm159@pusan.ac.kr (S.M.P.); ckim@pusan.ac.kr (C.-S.K.); 3 Department of Cogno-Mechatronics Engineering, Pusan National University, Busan 46241, Republic of Korea; kgmin01@pusan.ac.kr; 4 The Crystal Bank Research Institute, Pusan National University, Busan 46241, Republic of Korea

**Keywords:** FMCW LiDAR, spectral multiplexing, unambiguous ranging

## Abstract

Frequency-modulated continuous-wave (FMCW) light detection and ranging (LiDAR) based on coherent ranging is a technology capable of high-resolution distance measurement while remaining robust against ambient light interference. However, extending the measurable range remains challenging due to (i) the coherence length limitation of the laser and (ii) distance ambiguity caused by frequency ambiguity in coherent detection. To overcome these limitations, we propose an unambiguous range-extended FMCW LiDAR enabled by arrayed waveguide grating (AWG)-based spectral multiplexing. By spectrally demultiplexing the reference arm into four wavelength channels with sequentially designed optical path delays, multiple independent interference signals are obtained simultaneously without increasing the number of photodetectors or optical couplers. A channel-pair-based distance decoding algorithm is further introduced to resolve distance ambiguity by classifying detection outcomes across adjacent channels and selectively applying predefined operations. The proposed FMCW LiDAR system effectively extends the measurable range to approximately five times that of a conventional FMCW LiDAR. Experimental results demonstrate high measurement accuracy and successful reconstruction of three-dimensional distance maps, validating the system’s potential for extended-range FMCW LiDAR applications.

## 1. Introduction

Recently, light detection and ranging (LiDAR) technology has emerged as a core optical sensing method capable of realizing high-resolution three-dimensional (3D) spatial perception. It plays a crucial role in various applications such as autonomous driving, robotics, and machine vision [1,2,3,4,5,6]. The time-of-flight (ToF) LiDAR, which measures distance based on the propagation time of laser pulses, has been widely adopted due to its simple structure and ease of implementation [7,8,9]. However, the ToF LiDAR, which relies on direct detection of optical intensity, is highly susceptible to ambient light interference and exhibits a low signal-to-noise ratio (SNR) [10]. To overcome these limitations, frequency-modulated continuous-wave (FMCW) LiDAR has been actively investigated. FMCW LiDAR is a coherent ranging technology that uses optical interference, simultaneously providing high SNR and excellent immunity to ambient light compared with the ToF method [11,12,13,14]. Owing to these advantages, FMCW LiDAR has emerged as a promising next-generation long-distance mapping technology [15,16].

However, to extend the measurable range for long-distance measurements, FMCW LiDAR must overcome several limitations. First, the maximum measurable range is limited by the coherence length of the laser [17,18]. Second, in conventional FMCW LiDAR, the photodetector cannot distinguish between positive and negative frequency components. As a result, the beat spectrum becomes symmetric about the zero optical path difference (OPD) point, resulting in frequency ambiguity and hindering accurate frequency estimation. This frequency ambiguity directly translates into distance ambiguity, such that a target located at symmetric positions with respect to the zero-OPD point yields the same measured beat frequency and cannot be uniquely localized [19,20]. To overcome these limitations, various approaches have been actively investigated. Notably, differential detection schemes that generate a frequency-fixed carrier and a frequency-modulated subcarrier from a single laser have been reported to achieve kilometer-scale long-distance ranging beyond the intrinsic coherence length limitation of the laser [21]. Furthermore, methods employing symmetrical dual-sideband signals generated using an electro-optical phase modulator and optical band-pass filters have been proposed to suppress phase noise through differential processing, thereby improving long-distance measurement performance [22]. However, these differential detection-based approaches typically require additional hardware components, such as high-cost external modulators and complex RF signal-generation systems, which significantly increase system complexity and cost, thereby limiting scalability. Meanwhile, a stepped-delay interferometer (SDI) employing optical couplers (OCs) enables the formation of multiple reference paths for ambiguity resolution and range extension while maintaining a constant electrical bandwidth [23]. However, further range extension requires additional delay paths, which increases the number of photodetectors and OCs and consequently raises system complexity and cost. Therefore, a cost-effective architecture capable of generating multiple reference paths with only minimal additional hardware remains highly desirable.

Recently, wavelength-division devices, such as arrayed waveguide gratings (AWGs) and wavelength-division multiplexers (WDMs), have been increasingly adopted in LiDAR systems because they can efficiently separate optical channels. For example, a multi-beam single-photon LiDAR system has been realized by combining dense WDM and a blazed grating with sixteen laser diodes having equal frequency spacing, thereby generating multiple beams dispersed at equal angular intervals to enhance the field of view and scanning speed of the system [24]. In addition, a scan-less ToF LiDAR system has been implemented by demultiplexing a multi-wavelength laser into separate wavelength channels using WDM, dividing the optical paths, and employing a fiber array [25]. Accordingly, wavelength-division devices provide an attractive route to generate multiple effective optical paths in a compact and scalable manner.

In this study, the primary objective is to develop an unambiguous range-extended FMCW LiDAR system using AWG-based spectral multiplexing, enabling scalable range extension without a proportional increase in hardware complexity. To achieve this objective, we propose an FMCW LiDAR architecture in which the reference light of a Mach–Zehnder interferometer driven by a frequency-swept laser (FSL) is spectrally demultiplexed into multiple wavelength channels using an AWG. Each channel experiences a distinct and independent optical path, enabling the simultaneous generation and analysis of spectrally multiplexed interference signals. Using these signals, the target distance is determined by classifying channel-specific detection combinations and selectively applying the corresponding predefined operations. This architecture resolves distance ambiguity, thereby overcoming the coherence length limitation and extending the measurable range beyond that of a single-channel FMCW LiDAR. In contrast to SDI architectures, in which extending the measurement range requires a linear increase in the number of optical couplers and photodetectors, the proposed AWG-based approach is distinguished by its ability to generate multiple reference paths in parallel within a single compact device. As a result, the hardware complexity and system cost do not scale proportionally with the number of reference arms, making the proposed architecture particularly well-suited for scalable and cost-effective long-range FMCW LiDAR implementations. To further fulfill the objective of validating the proposed approach, we conducted one-dimensional (1D) ranging and three-dimensional (3D) imaging experiments, demonstrating an approximately fivefold extension of the measurable range compared to a single-channel FMCW LiDAR. The accuracy and stability of the system were further confirmed through 100 repeated measurements.

## 2. Principle of AWG-Based Spectral-Multiplexing and Distance Decoding

**Figure 1:**
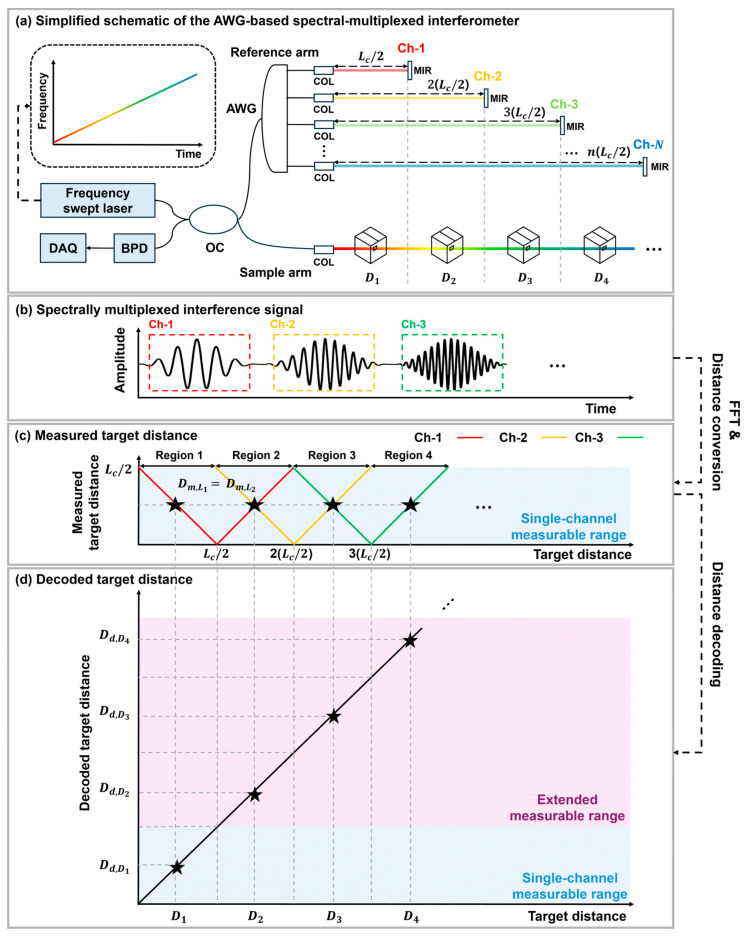
Principle of AWG-based spectral-multiplexing and distance decoding. (**a**) Simplified schematic of the AWG-based spectral-multiplexed interferometer. BPD: balanced photodetector, OC: optical coupler, COL: collimator, AWG: arrayed waveguide grating, DAQ: data acquisition, MIR: mirror. (**b**) Spectrally multiplexed interference signals dependent on the relative position of each mirror and the target. (**c**) Measured target distance with increasing actual target distance. (**d**) The decoded target distance increases with increasing actual target distance.

Figure 1 illustrates the operating principle of an unambiguous range-extended FMCW LiDAR enabled by AWG-based spectral multiplexing. Figure 1a depicts the multi-channel configuration where the reference light path is spectrally demultiplexed into N wavelength channels by the AWG. The optical signal emitted from the FSL is split into a sample arm and a reference arm by an OC. The OPD of the reference light for each channel is designed to increase sequentially by steps of $L_{c}/2$, where $L_{c}$ denotes the coherence length of the FSL. In this case, the maximum number of channels is N, and the number of configurable channels is determined by the sweep bandwidth of the FSL and the available AWG channel counts.

As the FSL sweeps in wavelength (or wavenumber *k*), the AWG passbands correspond to distinct wavelength intervals, and thus the interference contributions from different channels appear as separated segments in the measured time trace. Consequently, multiple independent interference signals are simultaneously obtained from different channels (Figure 1b). By applying a fast Fourier transform (FFT) to the interference signal obtained from each channel, the beat frequency $f_{b}$ can be extracted. The measured target distance $D_{m}$ is then calculated using Equation (1) [26,27].

(1) $$D_{m}=\frac{f_{b}cT}{2B}$$

where c denotes the speed of light, T is the sweep period, and B is the frequency sweep range. The measured distances for each channel are shown in Figure 1c. In a conventional FMCW LiDAR employing a single-channel reference arm, if distance ambiguity is not resolved, the measurable range is limited to $L_{c}/2$. This limitation arises because the photodetector cannot distinguish between positive and negative frequency components, leading to symmetric frequency responses about the folding point where the OPD becomes zero. For the n-th channel, this zero-OPD point is located at $n(L_{c}/2)$. Consequently, frequency ambiguity leads to distance ambiguity, making it impossible to determine the exact position of the target. For instance, if targets are located at $D_{1}$ and $D_{2}$ such that $\left|L_{c}/2-D_{1}\right|=\left|L_{c}/2-D_{2}\right|$, the measured target distances appear identical; that is, $D_{m,L_{1}}$ and $D_{m,L_{2}}$ cannot be distinguished. To resolve this distance ambiguity, we employ a distance decoding algorithm that reconstructs the unambiguous target distance by jointly interpreting measurements from adjacent AWG channels. This decoding enables unambiguous ranging beyond the coherence length-limited range of a single-channel reference arm, and the achievable measurable range scales with the number of AWG channels. The resulting decoded target distance is presented in Figure 1d. When the distance decoding is successfully performed across all channels, the measurable range is extended by a factor of $\left(N+1\right)$ compared to the single-channel measurable range.

**Figure 2:**
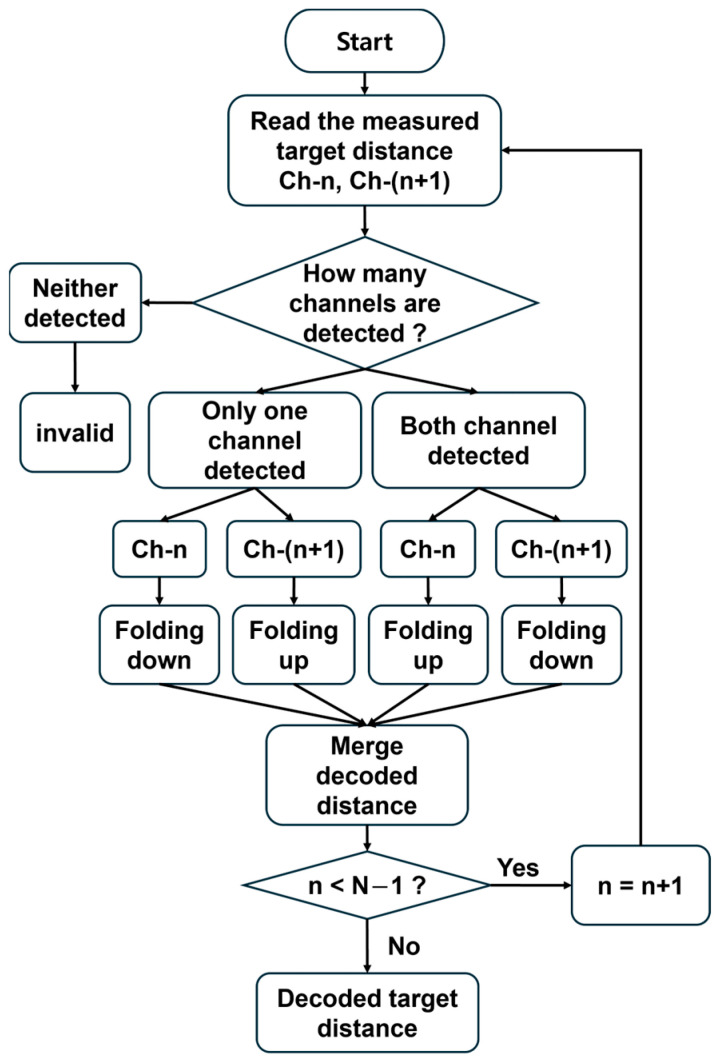
Flowchart of the distance decoding algorithm.

The detailed procedure of the distance decoding algorithm is illustrated in Figure 2. First, signal processing is performed on the measured target distance data using adjacent channel pairs, such as Ch-n and Ch-(n + 1). For each pair, the algorithm classifies the detection outcome based on whether the interference signal is undetected in both channels, detected in only one channel, or detected in both channels. Here, a signal is defined as detected when the amplitude of the corresponding FFT peak exceeds the noise floor by at least 25 dB. If the FFT peak amplitude does not surpass this threshold, the signal is regarded as undetected. This threshold ensures a sufficiently high SNR relative to the noise level, thereby preventing erroneous peak selection caused by noise fluctuations. If the signal is undetected in both channels, it is discarded as invalid. If a signal is detected, ‘folding down’ or ‘folding up’ operations defined in Equation (2) are selectively applied according to the corresponding case:

(2) $$D_{d}=\;\left\{\begin{array}{c} \;\;\;\;\;\;n(L_{c}/2)\;-\;D_{m}\;\left\|\;folding\;down\right.\\ n{(L}_{c}/2)\;+\;D_{m}\;\left\|\;folding\;up\right. \end{array}\right.$$

Specifically, when the signal is detected in only one channel, the operation is determined by which channel detects the signal. If the signal is detected in the preceding channel Ch-n, the ‘folding down’ operation is applied, whereas if it is detected in the succeeding channel Ch-(n +1), the ‘folding up’ operation is applied. For example, in the case of Ch-1 and Ch-2, this corresponds to Region 1 and 3 in Figure 1c. In contrast, if the signal is detected in both channels, the ‘folding up’ operation is applied to the preceding channel Ch-n, and the ‘folding down’ operation is applied to the succeeding channel Ch-(n + 1). For example, in the case of Ch-1 and Ch-2, this corresponds to Region 2 in Figure 1c. Once the calculations for each case are completed, the decoded distances are merged. If n < N − 1, the index is incremented to n + 1, and the process is repeated. Otherwise, the signal processing terminates, completing the distance decoding algorithm. Through this decoding process, the measurable range—which is otherwise limited by the laser coherence length—can be efficiently extended. To validate the proposed distance-decoding algorithm and the resulting range extension, we constructed an unambiguous range-extended FMCW LiDAR using an AWG and conducted a series of 1D ranging and 3D imaging experiments.

## 3. Experimental Setup and Results

### 3.1. Experimental Setup

**Figure 3:**
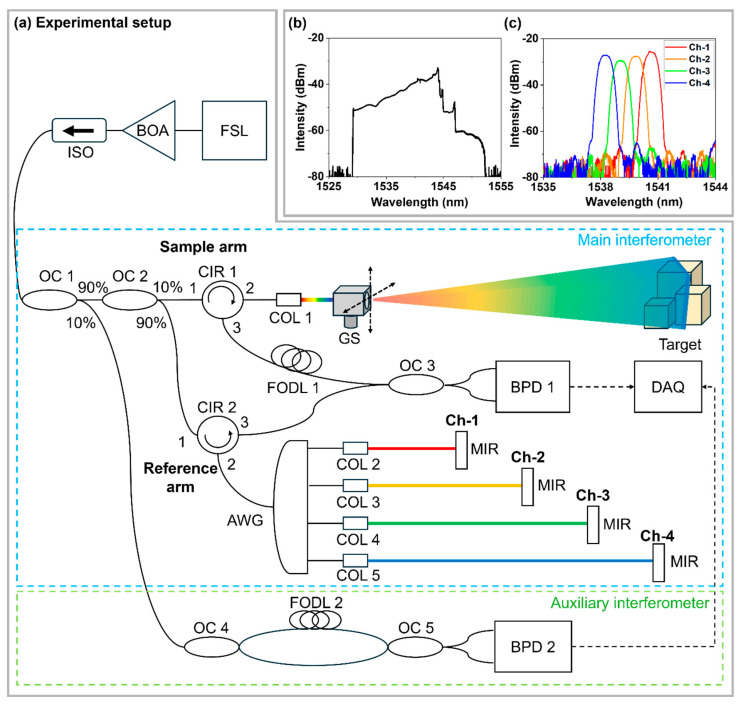
(**a**) Schematic of the unambiguous range-extended FMCW LiDAR employing AWG. FSL: frequency swept laser, BOA: booster optical amplifier, ISO: isolator, OC: optical coupler, CIR: circulator, COL: collimator, GS: Galvano scanner, BPD: balanced photo detector, DAQ: data acquisition, AWG: arrayed waveguide grating, FODL: fiber optic delay line, MIR: mirror. (**b**) Output spectrum of the FSL. (**c**) Optical spectra of the AWG.

Figure 3a illustrates the schematic diagram of the unambiguous range-extended FMCW LiDAR employing an AWG. The output generated by the FSL is amplified by a booster optical amplifier (BOA), passed through an optical isolator (ISO), and then split by a 90:10 OC 1. A 90% portion of the output is delivered to the main interferometer, where the light is further divided by OC2, which splits the optical power between the sample and reference arms (10:90). In the sample arm, the optical circulator (CIR 1) and the collimator (COL 1) guide the light and form a collimated beam. The galvanometer scanner (GS) scans the beam across the two-dimensional field, after which the backscattered light from the target is collected through COL 1 and CIR 1 and directed to OC 3. To compensate for the OPD introduced by the AWG, a fiber-optic delay line (FODL 1) is inserted between CIR 1 and OC 3. In the reference arm, the 10% output of the FSL is spectrally demultiplexed by the AWG into multiple wavelength channels, and the optical power is distributed across the channels accordingly. After passing through CIR 2, the demultiplexed light in each channel is collimated and reflected by a mirror (MIR), and the reflected light is recombined through COLs and OC 3 to generate an interference signal. The system is designed such that the zero-OPD point of Ch-1 corresponds to the 6-dB roll-off length $L_{c}/2$ of the point spread function (PSF). Each channel’s optical delay is designed sequentially according to its corresponding ${n(L}_{c}/2)$ position. The interference signals combined at OC 3 are converted into an electrical signal by a balanced photodetector (BPD 1). The converted electrical signals are digitized using a data acquisition (DAQ) system, allowing the interference signals from all channels to be recorded at a sampling rate of 500 MS/s during each sweep period.

The remaining 10% of the output split by OC 1 is directed to an auxiliary interferometer for k-linearization. In this auxiliary path, the light is divided into two paths by OC 4, with a 0.15 m FODL 2 inserted in one path to generate an interference signal used to estimate the nonlinearity of the FSL. The recombined signals at OC 5 generate an additional interference signal, which is converted into an electrical calibration signal by a BPD 2. Figure 3b illustrates the swept spectrum of the FSL (Optilab, Phoenix, AZ, USA, VCSEL-1544-T-SM) used in the experiment. The FSL exhibits a linewidth of 300 MHz, corresponding to a coherence length of approximately 0.32 m, and was operated with a sweep rate of 10 kHz with a sweep bandwidth of approximately 14.7 nm. Figure 3c displays the optical spectrum of the channels separated by the AWG (Agiltron, Woburn, MA, USA, AAWG). The center wavelengths of the AWG channels span the range from 1538.28 nm to 1540.64 nm, with an adjacent channel spacing of approximately 0.8 nm (100 GHz) and a full width at half maximum (FWHM) of about 0.75 nm for each channel. The employed AWG provides channel isolation exceeding 30 dB at the center wavelengths, ensuring sufficiently low inter-channel leakage. Although partial spectral overlap may occur near the passband edges, its impact on ranging accuracy was mitigated through signal processing. Specifically, the time samples corresponding to the channel-edge overlap region in each raw interferogram were excluded prior to FFT computation, and the FFT was performed using only the remaining samples. In addition, a Hanning window was applied to suppress sidelobe leakage within the band of interest, rendering the residual inter-channel crosstalk practically negligible in the measurements.

**Figure 4:**
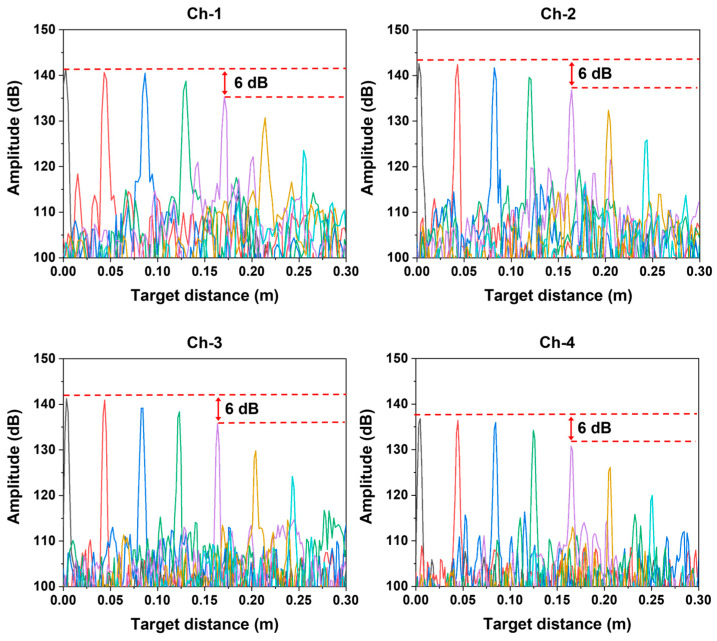
Point spread function (PSF) roll-off curves measured for each AWG channel at 0.04 m intervals up to 0.25 m.

Figure 4 shows the PSFs of each AWG channel for target distances measured up to 0.25 m using the unambiguous range-extended FMCW LiDAR. For all channels, the 6-dB roll-off point was measured at $L_{c}/2=0.17\;m$, indicating that the coherence length of the laser, $L_{c}$ is approximately 0.34 m. This value is consistent with the expected coherence length of approximately 0.32 m derived from the 300 MHz linewidth of the FSL. To ensure accurate distance measurements, k-linearization was performed using a resampling method using the proposed auxiliary interferometer [28,29]. The axial resolution measured at the $L_{c}/2$ position of each channel was approximately 5 mm for Ch-1 and 4 mm for Ch-2, 3, and 4. The discrepancy between the theoretical and experimental axial resolutions can be partly attributed to the application of windowing during the FFT process. The theoretical axial resolution determined by the center wavelength and FWHM of each AWG channel is approximately 1.4 mm. The discrepancy between the theoretical and experimentally measured axial resolutions ranges from approximately 2.6 mm to 3.6 mm. This discrepancy can be partly attributed to FFT windowing, which suppresses sidelobes at the expense of broadening the PSF. In addition, when using AWG-filtered channels, the effective bandwidth can be further reduced because the passband-edge overlap region is excluded prior to FFT processing, which also broadens the PSF. Moreover, residual k-linearization and dispersion mismatch may remain because the k-linearization is derived from a full-spectrum auxiliary interferometer, whereas the ranging interferograms are acquired with spectrally filtered AWG channels; this mismatch can manifest primarily as additional peak broadening [30,31].

### 3.2. 1D Measurement Results

**Figure 5:**
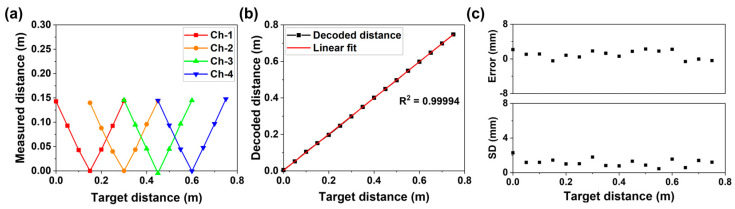
1D measurement results using the unambiguous range-extended FMCW LiDAR employing AWG. (**a**) Measured distance through each channel of the AWG from 0 m to 0.75 m. (**b**) Decoded distance using the distance decoding algorithm. (**c**) Error and standard deviation (SD) between the decoded distance and the target distance derived from 100 repeated measurements.

Figure 5 presents the 1D measurement results obtained using the unambiguous range-extended FMCW LiDAR. Figure 5a shows the measured target distance as a function of the actual target distance for each channel. Measurements were conducted at intervals of 0.05 m by translating the target across the sensing range and processing each channel’s interferogram independently. In the proposed system, each channel is activated sequentially in the wavelength domain, but in the time domain it is measured independently and continuously without any temporal switching between channels. As expected from the folded distance response in direct detection, each channel exhibits periodic folding (zero-OPD) points, and the measured distance repeats symmetrically about these points. The folding points for each channel appear at intervals of 0.15 m, ranging from 0.15 m to 0.6 m, because the OPD of the reference arm for each channel was designed to be slightly shorter than $L_{c}/2$ to maintain sufficient SNR during the experiments. The deviations in the OPD can shift the corresponding folding points; therefore, the folding point of each channel was experimentally calibrated. In the experiments, the folding point of each channel was determined by verifying the symmetry of the measured distance response with respect to the folding boundary. Specifically, the reference arm mirror was translated across the expected folding position using a linear stage with a resolution of 0.05 mm. The calibration was performed by confirming that the measured distances exhibited symmetric folding behavior around the folding boundary. The experimentally identified folding points were then used as reference boundaries for the subsequent distance decoding process. The average OPD error across all channels is approximately 1.27 mm, which is negligible. Figure 5b illustrates the relationship between the target distance and the decoded distance obtained using the distance decoding algorithm. The linear fitting results exhibit high linearity with a $R^{2}$ = 0.99994. Using the decoded results, the measurable range is extended to 0.75 m, which represents a fivefold increase compared to the 0.15 m range of a single-channel measurement without distance decoding. Figure 5c presents the error values and standard deviations derived from 100 repeated measurements at each target distance to evaluate measurement precision. The error was calculated by subtracting the actual target distance from the mean of 100 repeated measurements. Across all measured distances, the measurement error remains below 0.005 m, and the standard deviation is maintained below 0.004 m, demonstrating the high repeatability and accuracy of the proposed system.

### 3.3. 3D Measurement Results

**Figure 6:**
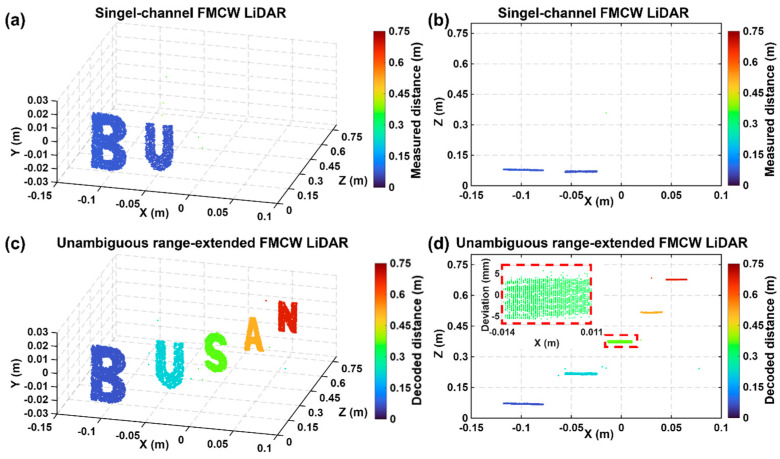
(**a**) Front view and (**b**) top view of 3D imaging results using single-channel FMCW LiDAR. (**c**) Front view and (**d**) top view of 3D imaging results using unambiguous range-extended FMCW LiDAR. The red dashed areas indicate the depth deviation of “S”.

Figure 6 presents the 3D imaging results obtained using the proposed unambiguous range-extended FMCW LiDAR. To verify the imaging performance of the system over its entire measurable range, targets consisting of letters “B”, “U”, “S”, “A”, and “N” cut from high-reflectivity sheets were placed at intervals of 0.15 m, spanning distances from 0.07 m to 0.67 m. A photograph of the targets is provided in Appendix A. Figure 6a shows the 3D imaging results reconstructed using a single-channel FMCW LiDAR without applying the distance decoding algorithm. The experiment was conducted with a field of view (FoV) of ±7.5° along the x-axis and ±2° along the y-axis, with a spatial sampling resolution of 950 × 200 pixels. The angular resolution is approximately 0.016° per pixel along the x-axis and 0.02° per pixel along the y-axis. The total acquisition time for a 3D image was approximately 19 s. While the depth profile acquisition rate is determined by the laser sweep rate of 10 kHz, the overall 3D imaging speed is currently scan-limited by the mechanical galvanometer scanners, which operate at a fast-axis (X-axis) frequency of 10 Hz and a slow-axis (Y-axis) frequency of 0.05 Hz. In the results, only targets falling within the channel’s effective (folded) measurement region are reconstructed, while targets outside that region are suppressed or mapped into neighboring folded intervals. Accordingly, as shown in Figure 6b, adjacent letters are observed at folded depth positions around the zero-OPD point in the single-channel FMCW LiDAR, where the distance decoding algorithm has not been applied. Figure 6c shows the reconstructed image at the actual target distances, obtained by combining the distance information from all channels using the proposed distance decoding algorithm. Figure 6d shows the top view of the reconstructed 3D image together with the depth deviation of the letter “S”. The results demonstrate that the letters are positioned at approximately 0.15 m intervals, consistent with the actual experimental configuration. In addition, the measured depth deviation remains within approximately 5 mm, which agrees well with the experimentally evaluated axial resolution. These results confirm that the proposed distance decoding algorithm enables accurate 3D reconstruction across the extended measurable range within the tested range, without depth misassignment or geometric distortion within the tested range.

## 4. Conclusions and Discussion

In this paper, we demonstrated an unambiguous range-extended FMCW LiDAR system based on AWG-based spectral multiplexing and a channel-pair distance decoding algorithm. By simultaneously acquiring spectrally multiplexed interference signals, the proposed architecture extends the measurable range without increasing hardware complexity. The distance ambiguity of single-channel FMCW LiDAR, caused by frequency ambiguity was resolved through channel classification and selective folding operations, enabling reconstruction of unfolded target distances beyond the coherence-length-limited range. Experimental results confirm the effectiveness of the proposed approach. 1D ranging experiments show a fivefold extension of the measurable range, from 0.15 m to 0.75 m, while maintaining high linearity (R^2^ = 0.99994), measurement errors below 0.005 m, and standard deviations below 0.004 m over 100 repeated measurements. 3D imaging experiments further verify accurate depth reconstruction across the extended range with consistent spatial resolution. In addition, the proposed distance decoding algorithm relies only on simple arithmetic operations, resulting in a low computational load. Compared with previously reported SDI-based FMCW LiDAR systems, which typically achieve range extensions of approximately three times that of a single-channel configuration, the proposed architecture achieves larger range expansion without requiring additional optical couplers or balanced photodetectors.

Although the present system successfully demonstrates range extension beyond the laser coherence length at laboratory-scale distances, several practical considerations remain for long-distance measurement. The current implementation requires multiple free-space reference paths corresponding to the number of AWG channels, resulting in a bulky configuration when extended to longer distances. In addition, long-distance measurements demand sufficient backscattered optical power, which may require optical amplification. Furthermore, the distance decoding algorithm is presently implemented in post-processing. In future work, the free-space reference arms will be replaced with fiber-type Faraday rotator mirrors to realize a compact all-fiber architecture that remains stable and practical even for extended measurement ranges. To secure sufficient optical power in the sample arm, optical amplifiers such as an erbium-doped fiber amplifier or a tapered semiconductor optical amplifier will be incorporated. These improvements will enable systematic evaluation under more realistic conditions, including low-reflectivity targets and rough surface scenarios. Moreover, for real-time operation, the distance decoding algorithm will be implemented on a field-programmable gate array, leveraging its parallel processing capability to perform FFT and decoding operations with low latency. This approach is expected to enable a more compact, cost-effective, and scalable system architecture suitable for practical long-range applications.

## Figures and Tables

**Figure A1:**
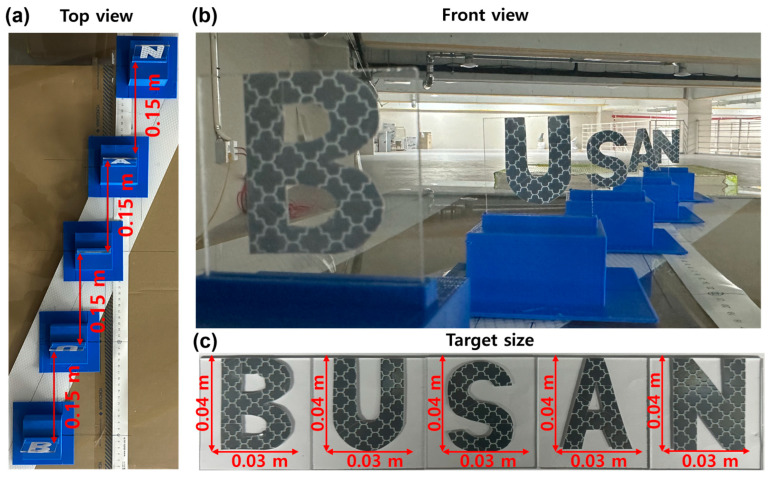
Photograph of 3D imaging target. (**a**) Top view of carved letters positioned at distances 0.07m, 0.22m, 0.37m, 0.52m, and 0.67m. (**b**) Front view of the target. (**c**) Dimensions of each letter used as the imaging target.

## Data Availability

Data will be available from the authors upon request.

## Appendix A

Figure A1a presents a top view of the target, where each letter is positioned at distances ranging from 0.07 m to 0.67 m with an interval of 0.15 m. Figure A1b shows a front view of the target, and the dimensions of each letter are 0.03 m in width and 0.04 m in height, as illustrated in Figure A1c.
